# Complete chloroplast genome sequence and phylogenetic analysis of *Ilex pernyi* Franch

**DOI:** 10.1080/23802359.2021.2002214

**Published:** 2021-11-26

**Authors:** Dan Wang, Chang Liu, Jin-jin Li, Dong-qin Guo, Hui-hui Du

**Affiliations:** a College of Biology and Food Engineering, Chongqing Three Gorges University, Chongqing, China; b Nanjing Institute for Comprehensive Utilization of Wild Plants, Nangjing, PR China

**Keywords:** *Ilex pernyi* Franch., complete chloroplast genome, phylogenetic analysis

## Abstract

*Ilex pernyi* Franch. is a Chinese unique medicinal plant with high value for decoration, human consumption, and as a medicine. In this study, we sequenced the complete chloroplast (cp) genome of *I. pernyi* Franch. and established its phylogenetic relationship in the Aquifoliaceae family. The total length of the chloroplast genome of *I. pernyi* Franch. was found to be 157,220 bp, with an overall GC content of 37.7% and a typical quadripartite structure with a pair of inverted repeats (IRs, 29,092 bp), which was separated by a small single-copy (SSC) region of 18,427 bp and a large single-copy (LSC) region of 89,609 bp. The cp genome contained 132 genes: 87 protein-coding, 37 tRNA, and 8 rRNA genes. The phylogenetic analysis indicated that *I. pernyi* Franch. was closely related to *Ilex cornuta*.

*Ilex pernyi* Franch. is an important genus of the *Ilex lineage* in Aquifoliaceae, which includes approximately 600 species distributed all over the world (Manen et al. 2010). Approximately 200 plants have been recorded in the *Flora of China* (Wu et al. 2020). Their main distribution is in the south of Qinling Mountains and the Yangtze River Basin. *I. pernyi* Franch. has high ornamental value, as well as edible and medicinal significance. Its leaves and fruits can be eaten or used as medicine, and the roots are utilized for decoction improving the body’s resistance and liver and kidney functions (Chen et al. 2008). According to the Guizhou herbal medicine and the Sichuan Traditional Chinese medicine records (1982 Edition), the needles of *I. pernyi* Franch. clear lung phlegm, relieve cough, and are used for the treatment of sore throat. However, most studies on this species have been focused on its chemical compositions (Xie et al. 2008, 2009), morphological properties (Feng 2020), whereas scarce molecular biology research has been conducted. Therefore, in this investigation we established the complete chloroplast (cp) genome of *I. pernyi* Franch. and revealed its phylogenetic relationships with closely related species or genera in the Aquifoliaceae.

Fresh leaf samples of *I. pernyi* Franch. were collected in Leshan, Sichuan Province, China (103ionships with closely related sMeanwhile, molecular material and voucher specimen (No. IPF001) of *I. pernyi* Franch. were collected and deposited at the Kunming Zhifen Biotechnology Co. Ltd. The total genomic DNA was extracted from fresh leaves by using the DNAsecure Plant Kit (TIANGEN, China) according to the manufacturer’s instructions, and sequenced with Illumina HiSeq 2500 (Novogene, Tianjing, China) platform with pair-end library (Cock et al. 2010). The raw data was filtered using Trimmomatic v.0.32 with default settings (Bolger et al.^2014^). Then paired-end reads of clean data were assembled into circular contigs using GetOrganelle (Jin et al. 2020) with *Ilex dabieshanensis* (NC_056201) as reference. Finally, the cpDNA was annotated by the Dual Organellar Genome Annotator GeSeq (Tillich et al. 2017) and CpGAVAS2 (Shi et al. 2019) The chloroplast genome was submitted to the GenBank (accession number: MZ464829).

The total length of the chloroplast genome was 157,220 bp, with 37.7% overall GC content. With typical quadripartite structure, a pair of IRs (inverted repeats) of 29,092 bp was separated by a small single-copy (SSC) region of 18,427 bp and a large single-copy (LSC) region of 89,609 bp. The cp genome was found to contain 132 genes, including 87 protein-coding genes, 37 tRNA genes, and 8 rRNA genes.

Based on the complete chloroplast genome of *I. pernyi* Franch. and 33 complete *Ilex* chloroplast genomes downloaded from NCBI, the phylogenetic tree of *Ilex* was constructed using *Gonocaryum lobbianum* and *Helwingia himalaica* as outgroups (Figure 1). The complete chloroplast genome sequences were aligned using MAFFT version 7 (Katoh and Standley 2013). A neighbor-joining (NJ) tree was then constructed using the MEGA v.7.0.26 (Kumar et al. 2016) with 1000 bootstrap replicates. The results showed that *I. pernyi* was most closely related to *I. cornuta*. In conclusion, the complete chloroplast genome in this study will promote further phylogenetic studies of the Aquifoliaceae family.

**Figure 1.**
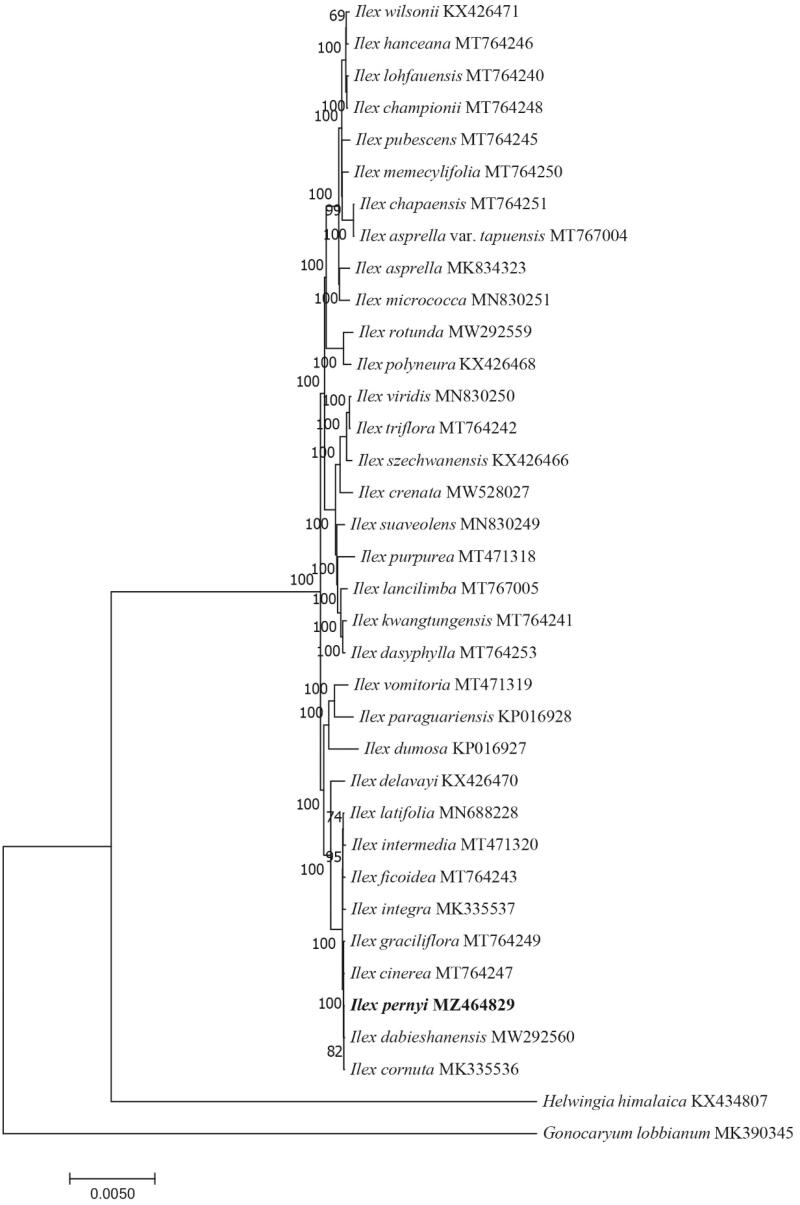
Neighbor-joining (NJ) phylogenetic tree of 34 species within the family Aquifoliaceae based on the complete chloroplast sequences using *Gonocaryum lobbianum* and *Helwingia himalaica* as an outgroup.

## Data Availability

The data that support the findings of this study are openly available in GenBank of NCBI at https://www.ncbi.nlm.nih.gov, MZ464829. The associated BioProject, SRA, and BioSample numbers are PRJNA759736, SRR15696126, and SAMN21193956, respectively.
